# Data Contamination in AI Evaluation

**DOI:** 10.2196/80987

**Published:** 2025-09-29

**Authors:** Alaeddin Acar

**Affiliations:** 1 Department of Neurosurgery, Kulu State Hospital Konya Turkey

**Keywords:** artificial intelligence, large language model, ChatGPT, emergency medicine, clinical performance examination, history taking, clinical reasoning, empathy, patient experience

This letter is regarding the recent publication of the article titled “Clinical Performance and Communication Skills of ChatGPT Versus Physicians in Emergency Medicine: Simulated Patient Study” by Park et al [1]. The study makes a significant contribution to the growing field of artificial intelligence (AI) evaluation in medicine, and I congratulate the authors on their valuable work. However, I would like to highlight a potential methodological limitation in the written examination portion of the study. The authors state that their examination questions were taken from a 2018 textbook, *100 Cases in Emergency Medicine and Critical Care* [2]. The AI model they tested, ChatGPT (OpenAI), was trained on huge amounts of public text from the internet, which likely included this textbook. This means ChatGPT may have seen exactly the same questions and answers during its training.

This problem is known as “data contamination.” If the AI has already seen the test questions, its high scores might show good memory, not good medical reasoning. This makes the comparison to human doctors, who were seeing the questions for the first time, unfair. The study found that ChatGPT performed much better than doctors on this written test, but this result could be due to this methodological limitation.

Other researchers in the field are aware of this problem and take steps to avoid it. For example, a study by Busch et al [3] on radiology used private, members-only cases that were not likely in the AI’s training data to minimize this risk. Another study by Noda et al [4] on a Japanese medical examination used questions from an examination that took place after the AI’s training data cut-off date.

These studies show the importance of using new and unseen questions when testing AI. Because the study by Park et al [1] did not use this approach, I believe the results of their written examination should be viewed with caution. Future studies must use methods like those in the Busch et al [3] and Noda et al [4] papers to ensure a fair and valid test of AI’s abilities.

## Abbreviations

AI: artificial intelligence
